# Phytochemistry, Pharmacology and Traditional Uses of Plants from the Genus *Trachelospermum* L.

**DOI:** 10.3390/molecules22091406

**Published:** 2017-08-24

**Authors:** Zefeng Zhao, Xirui He, Yuhui Zhao, Ying Sun, Xufei Chen, Ye Cun, Linhong Huang, Yajun Bai, Xiaohui Zheng

**Affiliations:** 1 Key Laboratory of Resource Biology and Biotechnology in Western China, Ministry of Education, Northwest University, 229 Taibai Road, Xi’an 710069, China; zzf598155752@sina.com (Z.Z.); xiruihe@163.com (X.H.); zyhzyh4115@yeah.net (Y.Z.); yingsun09@163.com (Y.S.); Chenxf@163.com (X.C.); cuanye2010@163.com (Y.C.); baiyj@nwu.edu.cn (Y.B.); 2 Honghui Hospital, Xi’an Jiaotong University, Xi’an 710054, China; 3 College of Chemistry and Materials Science, Northwest University, Xi’an 710069, China

**Keywords:** *Trachelospermum*, lignans, anti-inflammatory, analgesic, antitumor, review

## Abstract

This paper is intended to review advances in the botanical, phytochemical, traditional uses and pharmacological studies of the genus *Trachelospermum*. Until now, 138 chemical constituents have been isolated and characterized from these plants, particularly from *T. asiaticum* and *T. jasminoides*. Among these compounds, lignans, triterpenoids, and flavonoids are the major bioactive constituents. Studies have shown that plants from the genus *Trachelospermum* exhibit an extensive range of pharmacological properties both in vivo and in vitro, including anti-inflammatory, analgesic, antitumor, antiviral and antibacterial activities. In Traditional Chinese Medicine (TCM) culture, drugs that include *T. jasminoides* stems have been used to cure rheumatism, gonarthritis, backache and pharyngitis, although there are few reports concerning the clinical use and toxicity of these plants. Further attention should be paid to gathering information about their toxicology data, quality-control measures, and the clinical value of the active compounds from genus *Trachelospermum*.

## 1. Introduction

The genus *Trachelospermum* L., a member of the dobane family Apocynaceae and first described as a genus in 1851, is widely distributed in Asia and is particularly native to the subtropical regions . The general name of the genus is Caulis Trachelospermi. *Trachelospermum* is also called as “Luoshi/络石 (in Chinese)”, “rakusekito (u)/らくせきとう (in Japanese)”, “낙석등 (in Korean)”. The genus contains about 30 species, mainly distributed in tropical and subtropical zones. Ten species and six variant species are widespread in several provinces in China, especially the East and the Central South of China. The dried leaves and stems of the genus plants can be used as medicine. The stems of *Trachelospermum* plants have been used as local and traditional medicine in China, Japan and Korea, etc. (https://en.wikipedia.org/wiki/Trachelospermum). The species *T. jasminoides* and *T. asiaticum* are important medicinal sources. The dried stem part of *T. jasminoides* is recorded in various versions of Chinese Pharmacopoeia and is also used as a herbal medicine in Korea and Japan. In Traditional Chinese Medicine (TCM), the dried stem of *T. jasminoides* is used alone or mixed with other herbs for the treatment of rheumatism, blood cooling, gonarthritis, backache, pharyngitis and bruises diseases. The existing literature demonstrates that the therapy with the medicine is safe and effective both internally and externally. Plants of the genus *Trachelospermum* are enriched in multiple structurally diverse and biologically important lignans [1] and their glycosides, as well as triterpenoids [2] and flavonoids [3]. Modern pharmacognosy and pharmacology have revealed that lignans have strong antitumor activites [4]. To date, there are no published comprehensive reviews on the phytochemistry, traditional uses, pharmacology and toxicological information of plants from genus *Trachelospermum*. In this review, we summarize the progress on phytochemical studies over the past decades, with all the elucidated compounds listed. The biological characterization of the extracts or components isolated from this genus are also discussed. We believe this paper will be a guide for the full utilization of *Trachelospermum* plants for new drug development and pharmaceutical applications.

## 2. Botany

**Figure 1:**
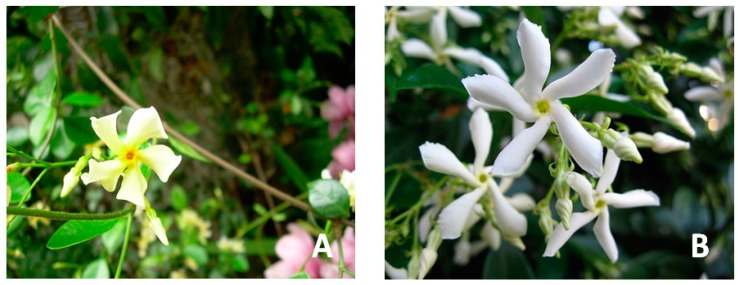
*Trachelospermum* plants: (A) *T. asiaticum* flowers and stems; (B) *T. jasminoides* flowers. (https://en.wikipedia.org/wiki/Trachelospermum).

The genus *Trachelospermum* belongs to the family Apocynaceae (Figure 1). *Trachelospermum* plants pertain to lianas, which usually grow to a height of 10 m. The lianas are woody, stems are brownish and lenticellate, and leaves are blade ovate or narrowly elliptic. The petiole grows from 2 to 12 mm long. The flowers are normally white or purplish, 5-merous, with the small and deeply divided calyx, and 5–10 basal glands inside, apices are usually denticulate. The flowering stages range from March to July, and the fruit phases are normally from July to December [5].

## 3. Phytochemistry

**Figure 2:**
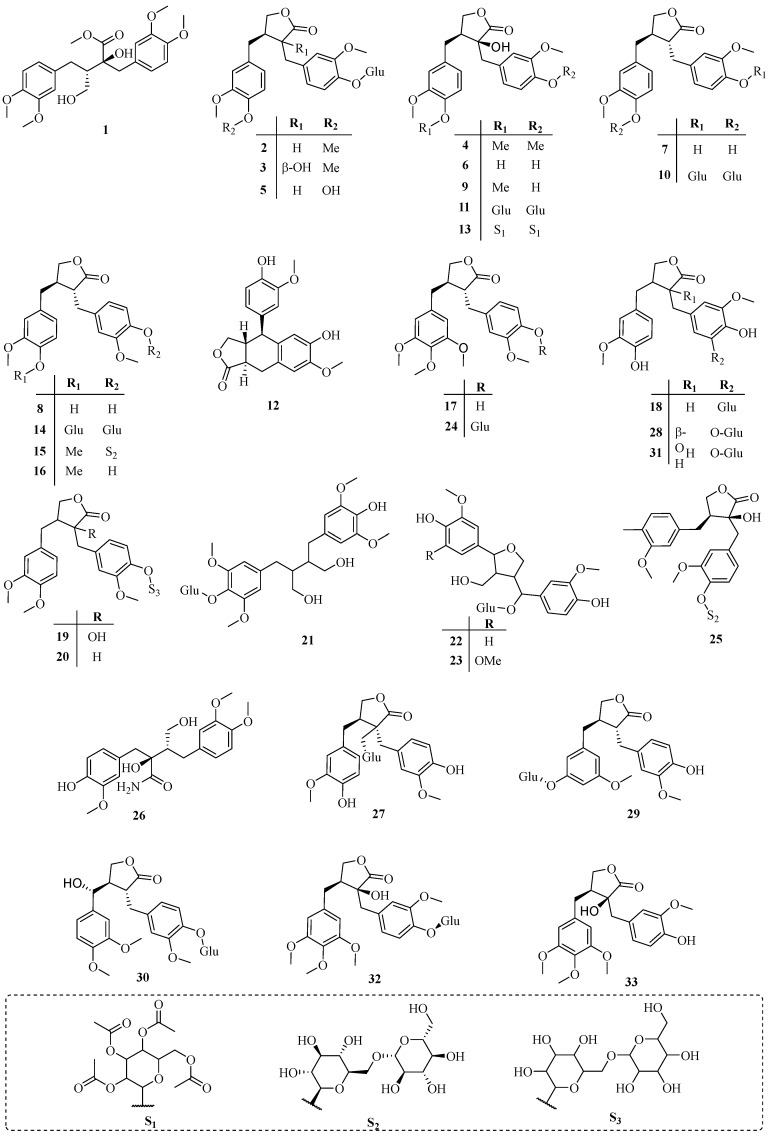
Lignans from the genus *Trachelospermum*.

**Figure 3:**
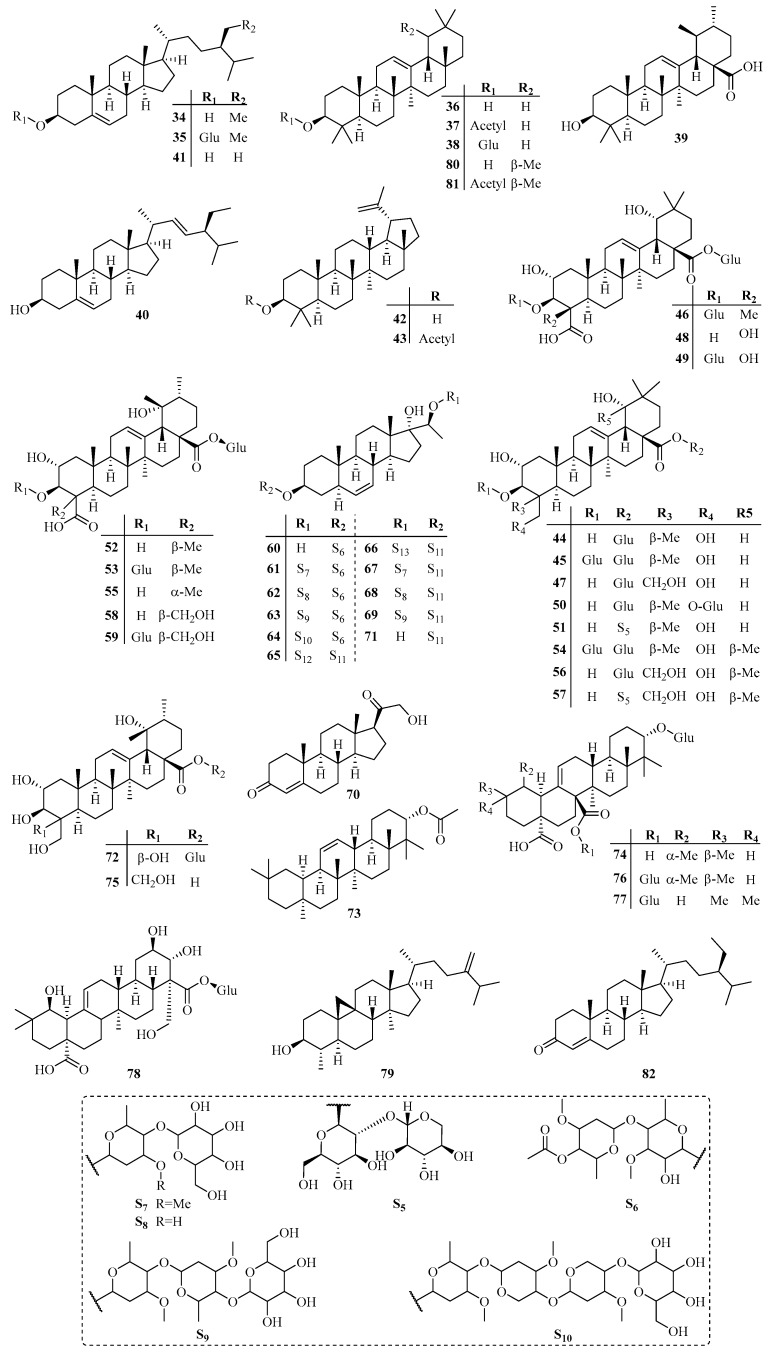
Triterpnoids from the *Trachelospermum* plants.

**Figure 4:**
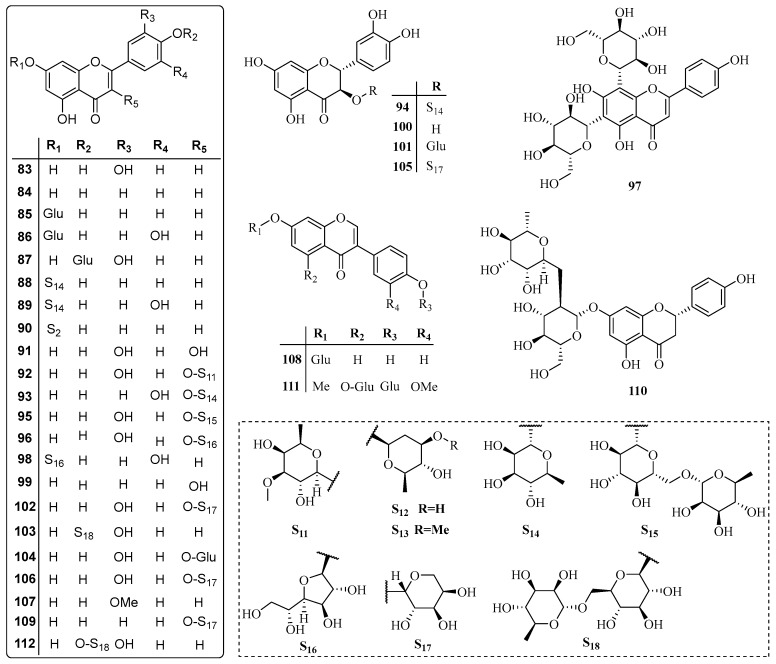
Flavonoids from the *Trachelospermum* plants.

**Figure 5:**
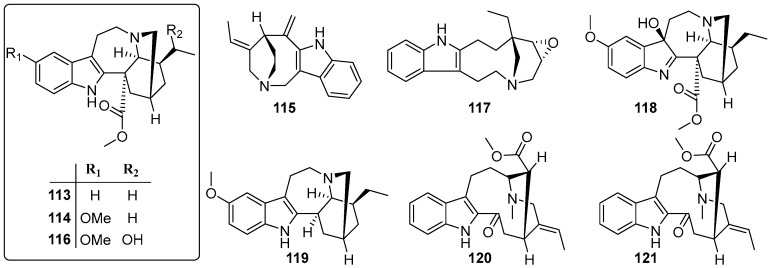
Alkaloids from the *Trachelospermum* plants.

**Figure 6:**
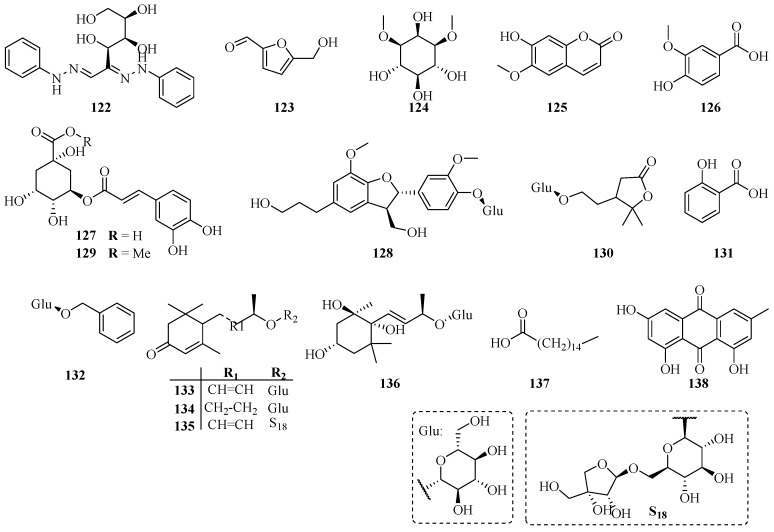
Other compounds from the *Trachelospermum* plants.

The species *T. jasminoides* (Lindl.) Lem. and *T. asiaticum* (Siebold & Zuccarini) Nakai are the most extensively studied species of the genus *Trachelospermum*. Besides, *T. liukiuense*, *T. axillare*, *T. lucidum, T. fragrans*, *T. difforme* and *T. gracilipes* are also used for phytochemistry research. Up to now, over 130 compounds have been isolated from the genus *Trachelospermum(*Table 1*)*, among which lignans (23.9%) [2,6,7,8,9,10,11,12,13,14,15,16,17,18,19,20], triterpenoids (35.5%) [2,21,22,23,24,25,26,27,28,29,30,31], flavonoids (21.7%) [13,21,32,33,34,35,36,37,38,39,40,41,42] are the major categories. All the compounds we summarize are compiled in Table 1. The specific structures of the compounds are drawn in Figure 2, Figure 3, Figure 4, Figure 5 and Figure 6.

### 3.1. Lignans

**Figure 7:**
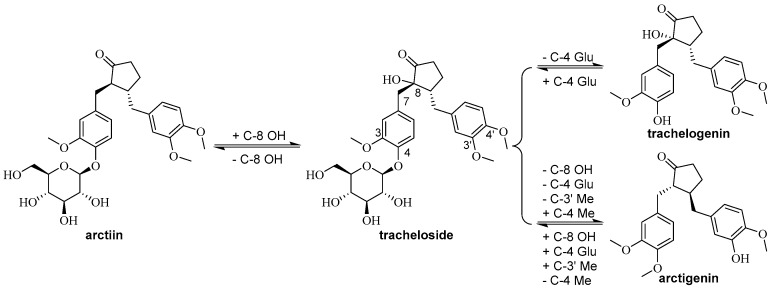
Transformation of representative lignans from *Trachelospermum*.

Lignans are the representative and predominated type of compounds from the genus *Trachelospermum*, and are valuable resources for drug design. So far, 33 lignans have been isolated from *Trachelospermum* plants. In this cluster, dibenzyltyrolactones are the principal compounds, while dibenzylbutane (compounds **1**, **21**, **26**) and arylnaphthalene (compound **12**) lignans have been isolated as well. Therefore, according to the Chinese Pharmacopoeia, tracheloside (**3**) is a quality control standard for the genus *Trachelospermum* plants, and it is also an active constituent with antitumor [4], anti-estrogenic [1], and α-glucosidase inhibitory activities [48], among others. Besides, other active compounds including arctiin (**2**), trachelogenin (**9**) and arctigenin (**16**) are also widely researched (Figure 7).

### 3.2. Triterpenoids

Triterpenoids are also widely distributed in this genus. To date, 49 triterpenoids have been isolated from *T. asiaticum*, *T. jasminoides*, *T. liukiuense* and *T. fragrans.* The structures of these compounds include tetracyclic and pentacyclic triterpenoids. Tetracyclic triterpenoids are mainly of the lanostane type with β-H on C (8), C (10) and C (13), α-H on C (9). Saccharide chains are connected to the C (8) or C (25) positions. Pentacyclic triterpenoids are divided into oleanane (compounds **36**–**38**, **81**, **46**–**48**), ursane (compounds **39**, **52**, **53**, **55**, **58**, **59**, **72**, **74**–**76**, **80**), lupine (compounds **42**, **43**) and pregnane (compounds **60**–**69**) types. Among them, β-sitosterol (**34**) [2] and α-amyrin (**80**) [31] are the most extensively studied compounds. In addition, some of the triterpenoids are esterified with saccharide residues, including β-d-glucopyranosyl, α-l-arabinopyranosyl, β-d-galactopyranosyl and other complex glycan residues.

### 3.3. Flavonoids

Approximately 30 flavonoids have been isolated from the genus *Trachelospermum.* Flavonoids (compounds **83**–**90**, **98**, **103**, **107**, **112**), flavonols (compounds **91**–**93**, **95**, **96**, **99**, **102**, **104**, **106**, **109**), isoflavones (compounds **108**, **111**) and anthocyans (compounds **94**, **100**, **101**, **105**) can be found in this class of compounds, among which flavonoids and flavonols predominate. Biologically important flavonoids such as luteolin (**83**), apigenin (**84**), quercetine (**91**), eldrin (**95**), kaempferol (**99**) and naringin (**110**) are included in these compounds. It is considered that the antioxidant activity [3] and anticancer effect [42] of *Trachelospermum* is related to the flavonoids.

### 3.4. Alkaloids

Alkaloids are less widespread in *Trachelospermum* plants. Up to now, only nine of this class of compounds have been isolated from *T. jasminoides*, with skeletons of the monoterpenoid indole type. Bioactivity research on *Trachelospermum* alkaloids is still rare [43,44].

### 3.5. Other Compounds

In addition to the compounds above, some compounds are also widely found in these medicinal plants such as 5-hydroxymethylfuraldehyde (**123**), scopoletine (**125**), vanillic acid (**126**), chlorogenic acid (**127**), salicylic acid (**131**) and emodin (**138**), etc. Besides, essential oils also have been separated form *Trachelospermum* plants, *E*-nerolidol, α-phellandrene and *trans*-linalool are the major ingredients of these essential oils [49].

## 4. Traditional Uses

Because of their versatile biological and pharmacological activities, *Trachelospermum* plants have been traditionally used for the treatment of rheumatism, blood cooling, gonarthritis, backache, pharyngitis and bruises. In TCM culture, *T. jasminoide* is dewcribed as bitter in taste, a little cold in nature and attributive to the liver and kidney meridians [50]. The traditional method of *T. jasminoide* consumption is usually decocting with water or wine. Studies on the side effects and safety evaluations of *T. jasminoide* are limited, although it is widely used in TCM. The Chinese Pharmacopoeia recommends a dose of 6–12 g for *T. jasminoide* [50].

*T. jasminoide* was listed for medicinal uses firstly in “*Sheng Nong′s herbal classic* (神农本草经)” during the Han Dynasty more than one thousand years ago. According to “*Ben Cao Gang Mu* (本草纲目)” (Ming Dynasty), the effect of *T. jasminoide* is mild, and the main function of *T. jasminoide* is to treat swelling and pain of bones and joints. Based on “*De Pei Ben Cao* (得配本草)” (Qing Dynasty), *T. jasminoide* is used to treat sore throat combined with *Belamcanda chinensis* and *Gardenia jasminoides*. Moreover, *T. jasminoide* can be used combined with *Radix Ginseng, Wolfiporia cocos* to treat turbid urine. In addition, *T. jasminoide* can treat tuberculosis, snake venom and haemorrhage with the combination of *Melastoma dodecandrum*, accordance with “*Jiang Xi Cao Yao* (江西草药)”. *T. jasminoide* is used to make wine containing several vine plants including *Piper kadsura, Spatholobus suberectus*, *Acanthopanar gracilistμlus, Taxillus sutchuenensis* and *Chaenomeles sinensis* to resist rheumatism and arthritis.

The preparations related to *Trachelospermum* recorded in the Chinese Pharmacopoeia are *She Xiang Kang Shuan* capsule and *Zhong Feng Hui Chun* pill, both of which are used for the treatment of dizziness, numbness and hemiparalysis caused by stroke [50].

## 5. Pharmacology

In recent years, the pharmacological activities of *Trachelospermum* have attracted attention. Modern research has shown that *Trachelospermum* plants play a role in anti-inflammatory and analgesic, antitumor and antiviral effects, although other pharmacology activities also have been documented.

### 5.1. Anti-inflammatory and Analgesic Activity

Plants in the genus *Trachelospermum*, especially *T. jasminoides* and *T. asiaticum*, have a long history of use in China as anti-rheumatic agents and for the treatment of arthritis-related diseases. In agreement with the traditional usage of plants from the genus *Trachelospermum*, several studies have illustrated that plants in this genus possess anti-inflammatory and analgesic effects both in vitro and in vivo. In 2003, ethanol extracts from several vine plants (*Spatholobus suberectus, Tripterygium wilfordii, Sinomenium acutum, Piper kadsura, T. jasminoides,* etc.) used in TCM to treat inflammatory conditions were evaluated against a panel of key enzymes relating to inflammation. The anti-inflammatory abilities against enzymes such as cyclooxygenase-1 (COX-1), cyclooxygenase-2 (COX-2), phospholipase A2 (PLA_2_), 5-lipoxygenase (5-LO) and 12-lipoxygenase (12-LO) were estimated. Among vine plants, *T. jasminoides* extract (extract/herbs = 0.85 g/30 g) exhibited significant anti-inflammatory ability against COX-1, COX-2, PLA_2_ and 12-LO with the IC_50_ values of 35, 138, 33 and 29 μg/mL respectively [51], which implied that *T. jasminoides* could prevent the generation of inflammatory mediators from arachidonic acid metabolism. Besides, the extract restrained the LPS-induced expression of inducible nitric oxide synthase (iNOS) protein and tumor necrosis factor-α (TNF-*α*) in RAW 264.7 cells in a dose-dependent manner (30–300 mg/mL) [52]. Meanwhile, the activating phosphorylations of p38 MAP kinase and NF-kB were inhibited by the extract in a dose-dependent manner, which were important for iNOS expression and NO production. Flavonoids were supposed to be contributors to the inflammatory effects of WET according to the HPLC analysis [3]. Compound **2** was identified by HPLC analysis and suggested to be the effective compound through exoteric experiments. Compound **35** isolated from the ethanol extract of *T. jasminoides* [53] was reported to be effective in reducing nitric oxide (NO) production in LPS-induced RAW 264.7 cells, in which inflammatory elements including TNF-*α* and interleukin 1β (IL-1β) were inhibited (inhibition values 23.3% and 64.1%).

In vivo, at a dose of 400 mg/kg, the *T. jasminoides* extract inhibited 12-*O*-tetradecanoylphorbol 13-acetate-induced mouse ear oedema, arachidonic acid-induced mouse ear oedema and acetic acid-induced writhing with the inhibition ratio of 65.7% and 61.8% inhibition ratio for oedema thickness and 45.8% inhibitory value for the writhing responses. The extract suppressed carrageenan-induced oedema in a dose-dependent and time-dependent manner in rats compared with the control groups [52].

Water extract of *T. jasminoides* (WET) showed antinociceptive effects in an acetic acid-induced writhing model, formalin test and carrageenan-induced edema. At a dose of 0.5 g/kg, WET performed markedly activity compared with control. Moreover, WET had significant inhibitory effects on TNF-α level, MDA level and activities of antioxidant enzymes such as SOD, glutathione peroxidase (GPx) and glutathione reductase (GRx) [3].

In the mouse model of type II collagen-induced arthritis (CIA), the mixture of ethanol extracts of *Trachelospermi caulis* and Moutan cortex radicis (TCMC) ameliorated histological deformation of joints and serum levels of rheumatoid arthritis biomarkers, such as cartilage oligomeric matrix protein, serum amyloid P and anti-collagen typeII IgG antibody. Additionally, TCMC suppressed pro-inflammatory cytokines (TNF-α, IL-1β and IL-6) and chemokines (macrophage inflammatory protein-1, monocyte chemoattractant protein-1) in CIA mice. Therefore TCMC repress the production of various inflammatory factors and the formation of osteoclasts through the inhibition of NF-kB and AP-1 activation [54].

### 5.2. Antitumor Activity

A herbal analgesic gel named *Tong-Luo-San-Jie* (TLSJ), was reported to be effective for alleviating bone cancer pain in rats. TLSJ is composed of ethanol extracts form the herbs *T. jasminoides*, *Piper kadsura*, *Dioscorea nipponica*, *Corydalis yanhuso*, etc. Walker 256 rat carcinoma cells were inoculated into Sprague-Dawley rats, then TLSJ was administered for 21 days. TLSJ treatment significantly restored bone cancer-induced decrease of paw withdrawal latency and mechanical threshold compared to inert gel. Meanwhile, it decreased the level of carboxyterminal pyridinoline cross-linked type I collagen telopeptides, bone-specific alkaline phosphatase and inhibited osteoclast activities in blood serum [55]. Furthermore, TLSJ was deemed valid in alleviating bone cancer-induced mechanical allodynia and thermal hyperalgesia by downregulating transient receptor potential channel expression in lumbar dorsal root ganglia and lumbar spinal cord interleukin-17A (IL-17A) in spinal astrocytes [56].

Dibenzyltyrolactone lignans were reported to show high inhibition on IFN-γ/STAT1 (signal transducer and activator of transcription) and IL-6/STAT3 pathway, which were considered to be important for anticancer action. At a concentration of 5 μM, compounds **9** and **16** exhibited distinct inhibition on IFN-γ/STAT1 (inhibition ratio values were 77.5% and 32.6%, respectively). Additionally, compounds **7**, **9** and **16** exhibited marked inhibition on IL-6/STAT3 (inhibition ratio values 89.8%, 96.1% and 44.2%, respectively). The activities of compounds **7**, **9** and **16** were further confirmed by IC_50_ evaluation. IC_50_ values for IFN-γ/STAT1 of compounds **9** and **16** were 3.14 μM and 9.46 μM and IC_50_ values for IL-6/STAT3 of compounds **7**, **9** and **16** were 2.92 μM, 3.63 μM and 6.47 μM [57], respectively.

Moreover, the cytotoxic activity compounds **7**, **9** and **16** were evaluated a1gainst human pancreatic cancer PANC-1 cells in nutrient-deprived medium. These compounds which have the (2*R*,3*R*)-absolute configuration exhibited preferential cytotoxicity compared with other lignan compounds in a concentration-dependent manner, with PC_50_ values of 0.54, 6.82 and 5.85 μM, respectively [58]. Compound **111**, a new isoflavonoid glycoside, was isolated from the ethyl acetate extract of *T. jasminoides*, which was found to have the inhibitory activity against HepG2 and HL-60 cancer cells with IC_50_ values of 131.5 and 58.2 μM, respectively [42].

### 5.3. Antiviral Activity

The inhibition of HIV-1 viral enzymes such as reverse transcriptase, HIV-1 protease andα-glucosidase were assessed using 18 herb extracts. Among these extracts, water extract and methanol extract of *T. asiaticum* showed weak inhibitive activity against HIV-1 protease, with the inhibition rate being 23.4% and 23.1% on the concentration of 100 μg/mL, respectively [59]. The ethanol extract of *T. jasminoides* and *T. liukiuense* showed weak anti-proliferative effect against T-cell lymphotropic virus type I (HTLV-I) in HTLV-I-infected T-cell lines. Extracts from *T. jasminoides* exhibited activities against MT-1 and MT-2 cells (EC_50_ values 10–100 μg/mL), while *T. liukiuense* only exhibited activity against MT-2 cells [60]. As for the effective ingredient, compound **9** isolated from *T. jasminoides* was recognised as a potential inhibitor of hepatitis C virus through interfering with the interactions between HCV glycoprotein E2 and the host entry factor CD81. Compound **9** inhibited cell culture-derived HCV and HCV pseudo-particles with the IC_50_ of 0.325 and 0.259 μg/mL [61].

### 5.4. Antibacterial Activity

The essential oil compositions of *T. jasminoides* obtained from Chiang Ra and Chiang Mai city were identified by GC-MS (Agilent Technologies, Palo Alto, CA, USA) analysis. More than 90 kinds of constituents were identified, with *E*-nerolidol, α-phellandrene (from Chiang Rai) and *trans*-linalool (from Chiang Mai) being the major components of the essential oil. The antibacterial activities of the essential oils were determined against Gram-negative and Gram-positive bacteria and minimum inhibitory concentration (MIC) values were evaluated. Essential oils from Chiang Rai showed a higher diameter of inhibition than that acquired form Chiang Mai, with the lowest MIC values against *E. aerogens* and *P. aeruginosa* are 7.81 (Chiang Rai) and 15.62 (Chiang Mai), respectively. Constituents from the essential oil such as *E*-nerolidol as α-phellandrene have been reported to exhibit antibacterial activity [49].

### 5.5. Other Biological Activities

In modern research, *Trachelospermum* plants were reported to have a variety of activities besides the abovementioned fields. Ethanol extract of *T. asiaticum* was considered to be effective on intestinal barrier function in intestinal epithelial cells. In a model of monolayers of intestinal epithelial cells (Caco-2), chloroform and ethyl acetate fractions promoted transepithelial electrical resistance (TEER) values and decreased quantity of permeated ovalbumin (OVA) flux. Compounds **3** and **9** were identified as the active constituents of this intestinal barrier function by HPLC analysis [62].

Additionally, antioxidant activities of essential oils of *T. jasminoides* were measured by the 1,1-diphenyl-2-picryl-hydrazyl (DPPH)radical scavenging property method (IC_50_ for Chiang Rai strain: 38.5, IC_50_ for Chiang Mai strain: 44.1 μg/mL) and ferric reducing antioxidant power (FRAP) assay (276.5 M Fe^2+^/g dry sample for Chiang Rai strain, 215.3 M Fe^2+^/g dry sample for Chiang Mai strain) [49].

Compounds **7**, **18**, **72** and **73** were isolated from the butanolic fraction of *T. lucidum*. Compounds **7** and **72** displayed moderate activity against lipoxygenase (LOX) as compared to positive control baicalein, with IC_50_ values of 9, 15.7 and 22.7 μg/mL respectively [26].

## 6. Conclusions

This review presents a comprehensive summary of the botany, phytochemistry, traditional uses and pharmacology of the genus *Trachelospermum*. To date, 138 compounds from *Trachelospermum* were characterized, and lignans, triterpenoids and flavonoids are the main compounds. The reports on the bioactivities of extraction and compounds from *Trachelospermum* are chronicled. Research on bioactive components are concentrated in lignans and flavonoids, which may contribute either directly or indirectly to the biological effects of the *Trachelospermum* genus.

There are multiple *Trachelospermum* species in the genus, with *T. jasminoides* and *T. asiaticum* being the two species that have received most attention due to their important medicinal value. Studies implemented through in vivo and in vitro experiments have demonstrated the bioactivities of *Trachelospermum* plants, most of which support their traditional medicine uses. Pharmacology studies are mainly focused on the anti-inflammatory and analgesic, antitumor and antiviral activity, and some preparations containing *T. jasminoides* have been developed into novel agents to prevent stroke and rheumatoid arthritis. However, the pharmacological effects of only a few components, such as compounds **3**, **7**, **9**, etc. have been studied. Precise investigations of the chemical composition of popular drugs are needed, as well as studies on the possible mechanism of action of the bioactive compounds and their structure-function relationships. Information on toxicity evaluations and randomized clinical trials for the genus *Trachelospermum* plant is very limited, although this plant is frequently used in TCM. Thus, further investigations on the toxicity, effective ingredients and clinical studies of species of the genus *Trachelospermum* are urgently needed to meet the requirements of evidence-based medicine.

## Figures and Tables

**Table 1:** Chemical constituents identified from the genus *Trachelospermum* L.

No.	Name	CAS	Source	Ref.
***Lignans***				
**1**	Methyl methyltrachelogenate	42320-76-3	A	[6]
**2**	Arctiin	20362-31-6	A	[7]
**3**	Tracheloside	33464-71-0	A	[7]
**4**	Methyltrachelogenin	33464-73-2	A	[7]
**5**	Matairesinoside	23202-85-9	A	[2]
**6**	Nortrachelogenin	34444-37-6	A	[8]
**7**	Matairesinol	580-72-3	A	[8]
**8**	Maculatin	25488-59-9	A	[8]
**9**	Trachelogenin	34209-69-3	A	[8]
**10**	Matairesinol 4,4′-di-*O*-β-d-glucopyranoside	38976-08-8	A	[9]
**11**	Nortrachelogenin 4,4′-di-*O*-β-d-glucopyranoside	38976-09-9	A	[9]
**12**	conidendrin	518-55-8	A	[10]
**13**	Nortrachelogenin-4,4′-di-*O*-β-d-glucopyranoside octaacetate	43179-85-7	A	[10]
**14**	2(3*H*)-Furanone,3,4-bis[[4-(β-d-glucopyranosyloxy)-3-methoxyphenyl]methyl]dihydro-, (3*R*-trans)-	41948-08-7	A	[11]
**15**	Arctigenin 4′-*O*-β-gentiobioside	41682-24-0	A	[12]
**16**	Arctigenin	7770-78-7	A	[13]
**17**	Traxillagenin	79288-73-6	A	[14]
**18**	Trachelosiaside	106647-12-5	A	[15]
**19**	Trachelogenin 4′-*O*-β-gentiobioside	106647-13-6	A	[15]
**20**	2(3*H*)-Furanone,3-[[4-[(6-*O*-β-d-glucopyranosyl-β-d-glucopyranosyl)oxy]-3-methoxyphenyl]methyl]dihydro-4-[(4-hydroxy-3-methoxy-phenyl)methyl]-, (3*R*-trans)-	106647-14-7	A	[15]
**21**	β-d-Glucopyranoside, 4-[4-(4-hydroxy-3,5-dimethoxyphenyl)-2,3-bis(hydroxymethyl)butyl]-2,6-dimethoxyphenyl	106647-15-8	A	[15]
**22**	Tanegoside A	131653-21-9	C	[16]
**23**	Tanegoside C	131653-22-0	C	[16]
**24**	Traxillaside	149415-62-3	D	[17]
**25**	2(3*H*)-Furanone,3-[[4-[(6-*O*-β-d-glucopyranosyl-β-d-glucopyranosyl)oxy]-3-methoxyphenyl]methyl]dihydro-3-hydroxy-4-[(3-methoxy-4-methylphenyl)methyl]-, (3*S*,4*S*)-	858127-40-9	B	[18]
**26**	Benzenebutanamide,α-hydroxy-α-[(4-hydroxy-3-methoxyphenyl)methyl]-β-(hydroxymethyl)-3,4-dimethoxy-, (α*S*,β*S*)-	132472-34-5	B	[18]
**27**	(3*S*,4*S*)-3-(β-d-Glucopyranosyloxy)dihydro-3,4-bis[(4-hydroxy-3-methoxyphenyl)methyl]-2(3*H*)-furanone	858127-38-5	B	[18]
**28**	(3*S*,4*S*)-3-[(3-β-d-Glucopyranosyl-4-hydroxy-5-methoxyphenyl)methyl]dihydro-3-hydroxy-4-[(4-hydroxy-3-methoxyphenyl)methyl]-2(3*H*)-furanone	858127-39-6	B	[18]
**29**	2(3*H*)-Furanone,4-[[3-(β-d-glucopyranosyloxy)-5-methoxyphenyl]methyl]dihydro-3-[(3-hydroxy-4-methoxyphenyl)methyl]-, (3*R*,4*R*)-	1069136-59-9	C	[19]
**30**	2(3*H*)-Furanone,4-[(R)-(3,4-dimethoxyphenyl)hydroxymethyl]-3-[[4-(β-d-glucopyranosyloxy)-3-methoxyphenyl]methyl]dihydro-, (3*R*,4*R*)-	1069136-60-2	C	[19]
**31**	2(3*H*)-Furanone,3-[[3-(β-d-glucopyranosyloxy)-4-hydroxy-5-methoxyphenyl]methyl]dihydro-4-[(3-hydroxy-4-methoxyphenyl)methyl]-, (3*R*,4*R*)-	1069136-62-4	C	[19]
**32**	2(3*H*)-Furanone,3-[[4-(β-d-glucopyranosyloxy)-3-methoxyphenyl]methyl]dihydro-3-hydroxy-4-[(3,4,5-trimethoxyphenyl)methyl]-, (3*S*,4*S*)-	1321810-65-4	B	[20]
**33**	(3*S*,4*S*)-Dihydro-3-hydroxy-3-[(4-hydroxy-3-methoxyphenyl)methyl]-4-[(3,4,5-trimethoxyphenyl)methyl]-2(3*H*)-furanone	1321810-66-5	B	[20]
***Triterpenoids***				
**34**	β-Sitosterol	83-46-5	A	[2]
**35**	Eleutheroside A	474-58-8	A	[2]
**36**	β-Amyrin	559-70-6	A	[2]
**37**	β-Amyrin acetate	1616-93-9	A	[2]
**38**	Ursolic acid	77-52-1	F	[21]
**39**	Teikaside A	77369-82-5	A	[22]
**40**	Stigmasterol	83-48-7	B	[23]
**41**	Campesterol	474-62-4	B	[23]
**42**	Fagarasterol	545-47-1	B	[23]
**43**	Lupenyl acetate	1617-68-1	B	[23]
**44**	Arjunglucoside I	62319-70-4	A	[24]
**45**	3-*O*-β-d-Glucopyranosyl-2α,3β,19α,23-tetrahydroxyolean-12-en-28-oic acid 28-*O*-β-d-glucopyranosyl ester	82843-99-0	A	[24]
**46**	Trachelosperoside F 2	109742-49-6	A	[24]
**47**	Trachelosperoside E 1	109742-50-9	A	[24]
**48**	Trachelosperoside D 1	109742-52-1	A	[24]
**49**	Trachelosperoside D 2	109742-51-0	A	[24]
**50**	Arjungenin 23,28-bis-*O*-glucopyranoside	109792-80-5	A	[24]
**51**	Olean-12-en-28-oic acid, 2,3,19,23-tetrahydroxy-,2-*O*-β-d-xylopyranosyl-β-d-glucopyranosyl ester, (2α,3β,4α,19α)-	109792-81-6	A	[24]
**52**	Suavissimoside F 1	95645-51-5	A	[25]
**53**	Urs-12-ene-23,28-dioic acid,3-(β-d-glucopyranosyloxy)-2,19-dihydroxy-, 28-β-d-glucopyranosyl ester, (2α,3β,4α)-	109825-38-9	A	[25]
**54**	3-*O*-β-d-Glucopyranosyl-2α,3β,19α,23-tetrahydroxyurs-12-en-28-oic acid 28-*O*-β-d-glucopyranosyl ester	82843-98-9	A	[25]
**55**	Trachelosperoside A 1	109750-36-9	A	[25]
**56**	Trachelosperoside B 1	109742-56-5	A	[25]
**57**	Trachelosperoside B 2	109742-55-4	A	[25]
**58**	Trachelosperoside C 1	109742-54-3	A	[25]
**59**	Trachelosperoside C 2	109744-39-0	A	[25]
**60**	Teikaside C 0	120727-46-0	A	[23]
**61**	Teikaside C IIa	120727-47-1	A	[26]
**62**	Teikaside C IIIa	120768-72-1	A	[26]
**63**	Teikaside C IVa	120727-49-3	A	[26]
**64**	Teikaside C IIc	120727-48-2	A	[26]
**65**	Teikaside A-Ia	114892-50-1	A	[27]
**66**	Teikaside A-Ib	114892-51-2	A	[27]
**67**	Teikaside A-IIa	114912-34-4	A	[27]
**68**	Teikaside A-IIc	114892-53-4	A	[27]
**69**	Teikaside A-IIIb	114892-54-5	A	[27]
**70**	Deoxycortone	64-85-7	C	[28]
**71**	β-d-Galactopyranoside, (3β,5α,20*S*)-17,20-dihydroxypregn-6-en-3-yl 6-deoxy-3-*O*-methyl-	77369-84-7	C	[28]
**72**	Leucioside	898801-61-1	E	[29]
**73**	Olean-11-en-3-ol, acetate, (3β)-	898798-42-0	E	[29]
**74**	Quinovic acid 3-*O*-β-d-glucoside	79955-41-2	B	[30]
**75**	Trachelosperogenin B	109742-53-2	B	[30]
**76**	Quinovic acid 3-*O*-β-d-glucopyranoside 27-*O*-β-d-glucopyranosyl ester	117751-62-9	B	[30]
**77**	Cincholic acid 3-*O*-β-d-glucopyranoside 27-*O*-β-d-glucopyranosyl ester	1004519-37-2	B	[30]
**78**	Trachelosperoside F	1004519-89-4	B	[30]
**79**	Cycloeucalenol	469-39-6	B	[31]
**80**	α-Amyrin	638-95-9	B	[31]
**81**	α-Amyrenyl acetate	863-76-3	B	[31]
**82**	β-Sitostenone	1058-61-3	B	[31]
***Flavonoids***				
**83**	Luteolin	491-70-3	A	[13]
**84**	Apigenin	520-36-5	A	[13]
**85**	Apigenin 7-glucoside	578-74-5	A	[13]
**86**	Luteolin 7-*O*-glucopyranoside	5373-11-5	A	[13]
**87**	Luteolin 4′-*O*-β-d-glucopyranoside	6920-38-3	A	[13]
**88**	Rhoifolin	17306-46-6	A	[32]
**89**	Luteolin 7-β-neohesperidoside	25694-72-8	A	[32]
**90**	Apigenin 7-*O*-β-gentiobioside	50826-94-3	A	[32]
**91**	Quercetin	117-39-5	F	[21]
**92**	Quercetin 3-β-galactoside	482-36-0	F	[21]
**93**	Quercimelin	522-12-3	F	[21]
**94**	Taxifolin 3-*O*-rhamnoside	29838-67-3	F	[21]
**95**	Eldrin	153-18-4	G	[33]
**96**	Quercetin 3-*O*-β-d-glucofuranoside	21637-25-2	G	[33]
**97**	Vicenin	23666-13-9	B	[33]
**98**	Luteolin 7-*O*-β-gentiobioside	70855-41-3	B	[33]
**99**	Kaempferol	520-18-3	B	[34]
**100**	Taxifolin	480-18-2	B	[35]
**101**	Taxifolin 3-*O*-β-d-glucopyranoside	27297-45-6	B	[35]
**102**	Quercetin *O*-arabinoside	30370-87-7	B	[35]
**103**	Apigenin-7-*O*-β-d-rutinoside	552-57-8	H	[36]
**104**	Quercetin 3-*O*-β-d-glucoside	482-35-9	D	[37]
**105**	Taxifolin 3-*O*-β-d-arabinopyranoside	209005-26-5	B	[38]
**106**	4*H*-1-Benzopyran-4-one,3-(β-d-arabinopyranosyloxy)-2-(3,4-dihydroxyphenyl)-2,3-dihydro-5,7-dihydroxy-, (2*S*,3*S*)-	901123-12-4	B	[38]
**107**	Chrysoeriol	491-71-4	B	[39]
**108**	Daidzin	552-66-9	B	[39]
**109**	Afzelin	482-39-3	A	[40]
**110**	Naringin	10236-47-2	B	[41]
**111**	5-(β-d-Glucopyranosyloxy)-3-[4-(β-d-glucopyranosyloxy)-3-methoxyphenyl]-7-methoxy-4*H*-1-benzopyran-4-one	1620385-26-3	B	[42]
**112**	Luteolin-4′-*O*-rutinoside	150460-69-8	B	[42]
***Alkaloids***				
**113**	Coronaridine	467-77-6	B	[43]
**114**	Voacangine	510-22-5	B	[43]
**115**	Apparicine	3463-93-2	B	[43]
**116**	19-epi-Voacangarine	6883-77-8	B	[43]
**117**	Conoflorine	15266-46-3	B	[43]
**118**	Voacangine-7-hydroxyindolenine	3464-63-9	B	[44]
**119**	Ibogaine	83-74-9	B	[44]
**120**	Vobasine	2134-83-0	B	[44]
**121**	Tabernaemontanine	2134-98-7	B	[44]
***Others***				
**122**	Glucosazone	4746-10-5	A	[6]
**123**	5-Hydroxymethylfuraldehyde	67-47-0	A	[45]
**124**	Dambonitol	523-94-4	A	[46]
**125**	Scopoletine	92-61-5	A	[14]
**126**	Vanillic acid	121-34-6	A	[14]
**127**	Chlorogenic acid	327-97-9	B	[35]
**128**	Dihydrodehydrodiconiferyl alcohol 4-*O*-β-d-glucopyranoside	131723-83-6	C	[16]
**129**	Methyl chlorogenate	29708-87-0	A	[40]
**130**	Trachelinoside	1251939-00-0	B	[47]
**131**	Salicylic acid	69-72-7	B	[47]
**132**	Benzyl glucopyranoside	4304-12-5	B	[47]
**133**	Roseoside A	54835-70-0	B	[47]
**134**	Icariside B_5_	114226-08-3	B	[47]
**135**	2-Cyclohexen-1-one,4-[(1*E*,3*R*)-3-[(6-*O*-d-apio-β-d-furanosyl-β-d-glucopyranosyl)oxy]-1-butenyl]-3,5,5-trimethyl-, (4*R*)-	143363-62-6	B	[47]
**136**	Actinidioionoside	540528-05-0	B	[47]
**137**	Palmitic acid	57-10-3	B	[31]
**138**	Emodin	518-82-1	B	[31]

Note: A: *T. asiaticum*; B: *T. jasminoides*; C: *T. liukiuense*; D: *T. axillare*; E: *T.* lucidum; F: *T. fragrans*; G: *T. difforme*; H: *T. gracilipes*.
